# Decision making and outcomes in colorectal cancer and frailty: the DeCaF study

**DOI:** 10.1308/rcsann.2025.0050

**Published:** 2025-07-15

**Authors:** A Kler, J Tay, C Slawinski, C Welch, S Moug, S Blackwell, R Arnott, P Mitchell, N Heywood

**Affiliations:** ^1^ University of Liverpool, UK; ^2^ Countess of Chester Hospital NHS Foundation Trust, UK; ^3^ Stockport NHS Foundation Trust, UK; ^4^ Lancashire Teaching Hospitals NHS Foundation Trust, UK; ^5^ King’s College London, UK; ^6^ NHS Greater Glasgow and Clyde NHS, UK; ^7^ NHS Golden Jubilee National Hospital, UK; ^8^ ACPGBI Patient Liaison Group, UK; ^9^ University of Oxford, UK

**Keywords:** Frailty, Colorectal cancer

## Abstract

**Introduction:**

Surgical resection is the main treatment for non-metastatic colorectal cancer (CRC). However, 6% of patients do not undergo surgery owing to frailty, according to the National Bowel Cancer Audit (NBOCA). The impact of preoperative evaluation and decision making on outcomes in frail patients is underexplored. This study examines variation in decision making for frail, older patients and the availability/use of resources by colorectal multidisciplinary teams (MDTs) across United Kingdom (UK) hospitals.

**Methods:**

A UK-wide questionnaire was distributed to colorectal MDTs via the NBOCA newsletter and social media (18 May to 30 June 2021). Part A assessed MDT structure and resource use; Part B explored MDT decisions for two simulated 75-year-old patients with colonic and rectal cancer.

**Results:**

Twenty MDTs responded. Decisions were MDT-driven in 55% (*n* = 11) and surgeon-driven in 45% (*n* = 9). Clinical examination (85%) and performance status (90%) were most used. Resource utilisation during MDT meetings varied across sites; for example, echocardiogram results were available and considered in MDT decision making in only 15% of centres. Cardiopulmonary exercise testing was used in 75%, anaesthetic assessment in 80%, frailty scoring in 25%, and preoperative geriatric assessment in 5%. Management of right-sided cancer was more consistent; rectal cancer decisions were more variable.

**Conclusions:**

Variation exists across MDTs in the availability and use of resources when managing frail CRC patients. There is less consensus for rectal than caecal cancer. These findings highlight the need for standardised MDT protocols to support equitable, patient-centred care in complex cases.

## Introduction

Colorectal cancer (CRC) is the fourth most common cancer in the UK, peaking between ages 70–74, a demographic increasingly affected by frailty.^1^ This overlap presents a critical challenge: balancing the benefits of surgical intervention against the risks and poorer postoperative outcomes often observed in frail patients.^2–4^

Although surgery remains the main curative option for CRC, frailty is an important consideration in surgical decision making. Although factors like age and comorbidities are known to influence clinician choices, less is understood about the specific impact of frailty and available resources during multidisciplinary team (MDT) discussions, despite these meetings being central to treatment planning.^5^

MDT meetings, introduced in the NHS in 1995, serve as the gold standard for cancer care, and often occur without all team members having assessed the patient directly.^6,7^ As a result, decisions frequently rely on objective data presented during the meeting. A common outcome, such as ‘assess fitness for surgery’, suggests further evaluation is needed after the MDT discussion.

However, the decision-making process remains intricate and multifaceted, with additional information required. Current National Institute for Health and Care Excellence (NICE) and British Geriatric Society (BGS) guidance advises that age should not a decisive factor when offering surgery; instead, a holistic approach should be adopted to consider the risks of surgery, patient preference, preoperative frailty status and postoperative needs such as geriatrician input.^8,9^ Yet, the presence of frailty is often under-recognised during MDT discussions, presenting a significant challenge in accurately assessing patients’ overall health status at this stage.

This study explored variation in MDT resources and decision making for patients with CRC, particularly in relation to patient frailty.

## Method

### Distribution of questionnaires and recruitment

United Kingdom (UK) colorectal MDTs were recruited between 18 May and 30 June 2021 via advertisement in the National Bowel Cancer Audit (NBOCA) newsletter through the Royal College of Surgeons, or via the study’s social media platform (X, formerly Twitter). MDTs expressed interest by contacting the study team through email or social media. No clinical trial coordinating centre was involved.

### Survey design

The survey design followed the CHERRIES checklist and included two parts.^10^ Part A was a site questionnaire assessing the MDT composition and access to preoperative assessment tools. Part B was presented as two simulated patients with newly diagnosed CRC. MDTs were instructed to discuss each patient as they would in a routine meeting and recommend treatment based on the provided information.

The two simulated patients had identical frailty levels and comorbidities, with the only difference being tumour location: one had a caecal tumour, the other a rectal tumour. The survey was constructed by the steering committee of this research group. The site questionnaire and the outline of the simulated patients, together with assessment and treatment options are described below. The survey included both multiple-choice and free-text fields to capture a range of responses, accounting for anticipated heterogeneity across MDTs. The survey was pilot tested on two colorectal consultant colleagues. No incentives were offered, and each section was limited to two pages. A maximum of 50 options were available per page. Respondents could revise answers before submission, but no changes were possible following submission.

### Site questionnaire

Participants were asked to indicate the number of surgeons performing CRC resections and the total number of CRC resections per year. Respondents were also asked to identify which healthcare professionals participated in MDT meetings, the extent of involvement of colorectal surgeons in patient decision making and treatment decisions, as well as the routine use of various clinical assessments including cardiopulmonary exercise testing (CPET). Lastly, the survey explored the use of geriatrician input. The full site questionnaire can be found in Figure S1 (available online).

### Simulated patients

#### Simulated patient 1 (caecal cancer)

##### History

Simulated patient 1 was a 75-year-old man with two months of altered bowel habit, weight loss and fatigue. Comorbidities included hypertension and osteoarthritis; he was a non-smoker with a body mass index of 27. Staging was T3 N0 M0, with no metastasis.

Functionally, he lived with his wife, who handled household tasks. He required a stick to walk, struggled with stairs and had an exercise tolerance of 100 yards. He left the house once a week with his wife, who usually drove.

##### Assessment of fitness for treatment

Options included clinical assessment, frailty score, Eastern Cooperative Oncology Group (ECOG) performance status, CPET, anaesthetic review, stress echocardiography, respiratory function, and referral to a geriatrician.

##### MDT recommendation

Based on the available information, the MDT had several treatment options to choose from, including resection of the primary colon cancer, consideration of a defunctioning stoma, transfusion, chemotherapy, radiotherapy or palliative care referral.

#### Simulated patient 2 (rectal cancer)

##### History

Simulated patient 2 had an identical history to the caecal cancer patient, except for tumour location: a mid-rectal tumour without evidence of metastasis, preliminarily staged as T3 N1 M0.

##### Assessment of fitness for treatment

The options to assess fitness for treatment were the same as provided for the caecal cancer patient.

##### MDT recommendation

Options included Hartman’s resection, anterior resection, defunctioning stoma, stenting (as definitive treatment), palliative radiotherapy, short-course radiotherapy and surgery, long-course chemoradiotherapy only, long-course chemoradiotherapy and surgery, chemotherapy or palliative care referral.

The questionnaires distributed to MDTs for the simulated patients is listed in Figure S2 (available online).

### Collation of questionnaires and analysis

Following completion of their questionnaires, participating MDTs subsequently sent responses via email, Google Forms or hard copies using a postal service. Study responses were randomised, collated and anonymised using Microsoft Excel. Completeness checks were performed manually. Only fully completed questionnaires were analysed. Response variables were reported using *n* (%) for categorical data and mean ± sd for continuous data. Graphs were constructed using GraphPad Prism 10.

### Ethics statement

The study was assessed against the HRA framework and deemed a service evaluation, no formal ethical approval was required.

## Results

### Respondents

**Figure 1:**
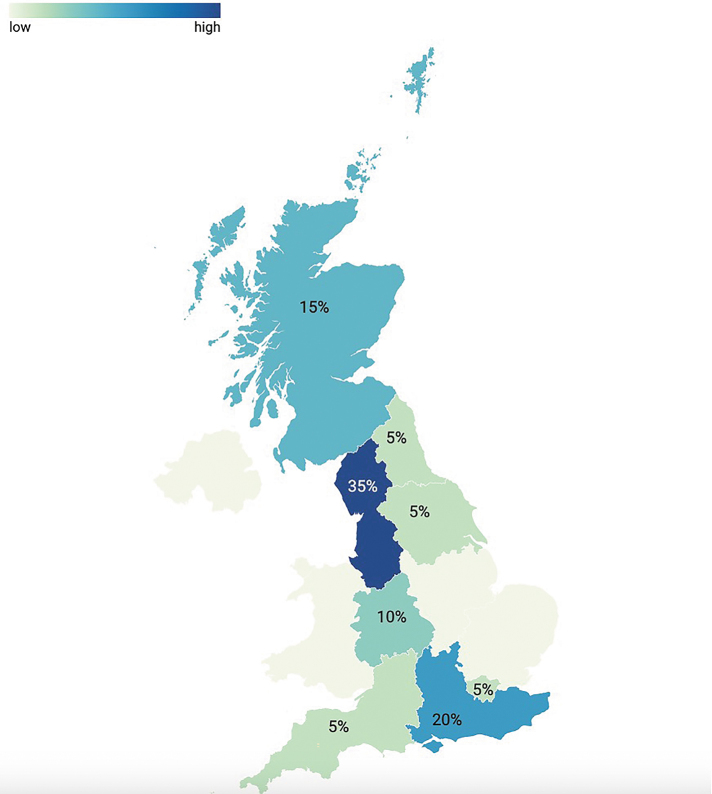
Heatmap of the different multidisciplinary team meetings responding to survey differentiated by UK region

Twenty-four centres responded. Four centres were excluded; two were international centres and thus ineligible for participation and two centres were excluded because they responded after the deadline. Therefore, 20 MDTs were included (Figure 1) with the highest representation (*n* = 7, 35%) coming from northwest England. A colorectal consultant acted as the respondent for each MDT. All returned questionnaires were fully completed.

### Site questionnaire

#### MDT composition

All MDTs had a surgeon, oncologist, pathologist and radiologist present. Only 5% of MDTs had a geriatrician attending. Figure S3a (available online) demonstrates the breakdown of attendees at the colorectal MDT.

#### Decision makers

In 55% of cases, the MDT made a definitive recommendation; in 45%, this was deferred to the surgeon pending clinical and other assessments.

#### Resources used in decision making

**Figure 2:**
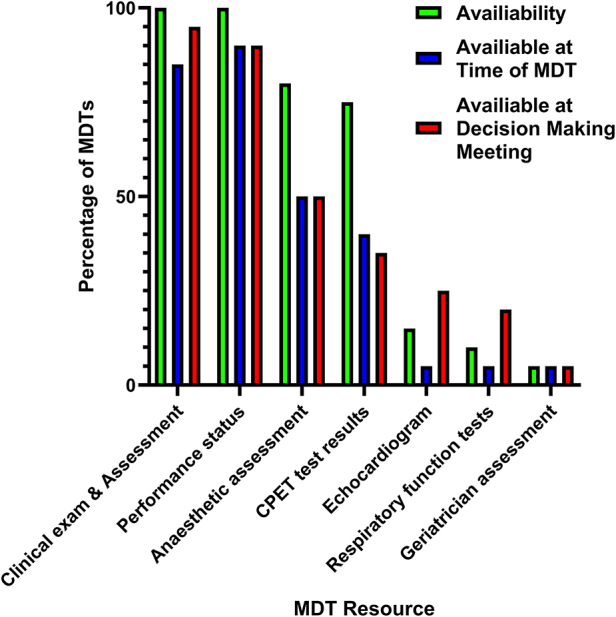
Availability of resources pertinent to multidisciplinary team meeting (MDT) decision making. Green bars indicate the general availability of the resource at the centre. Blue bars indicate the availability of investigation results at the time of the MDT meeting. Red bars indicate the availability of results by the time of the final decision-making meeting.

All MDTs made decisions based on clinical assessment and performance status. There was, however, variation in the availability of investigation results at the time of MDT meetings, which influenced whether they could be considered in decision making. These included anaesthetic assessments (80%), CPET (75%), respiratory function tests (15%) and echocardiograms (15%). A breakdown of when the resource results could be used is given in Figure 2.

Anaesthetic assessments were available to 80% of MDTs, but only 50% were completed by the time of decision making (Figure 2).

#### Scoring systems used in decision making

The World Health Organization (WHO) performance status was most commonly used (85%), whereas frailty scoring was used in the minority of MDTs (25%) (Figure S4 – available online).

#### CPET utilisation

**Figure 3:**
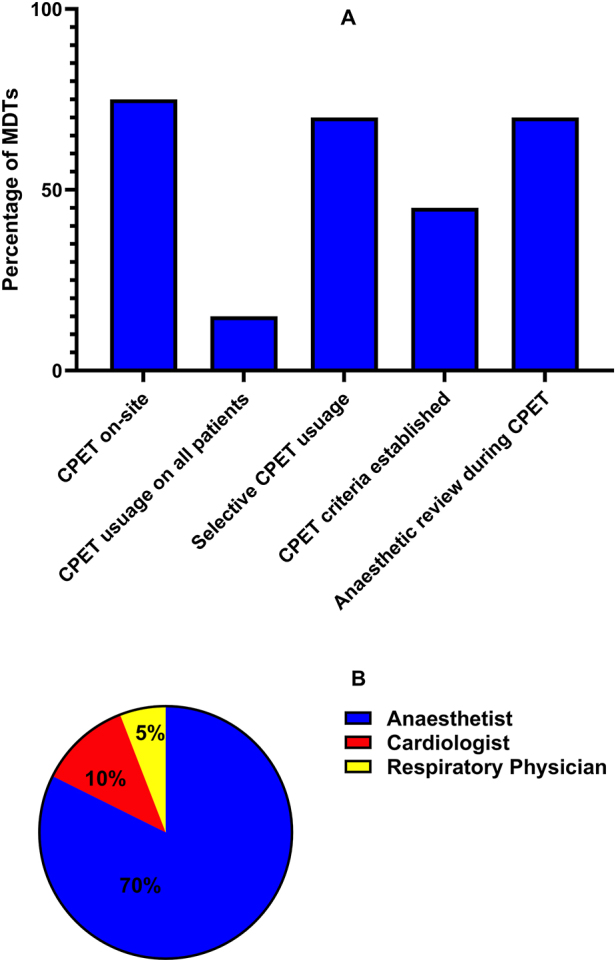
(a) Modality of cardiopulmonary exercise testing (CPET) use. (b) Identity of CPET administrator

Some 75% of centres routinely had CPET available. Most centres (70%) appointed an anaesthetist as the lead clinician responsible for overseeing the administration of CPET (Figure 3). Of these centres, 70% selectively use CPET; however, only 45% of centres employed a standardised pathway for it, and the remainder would request CPET in an ad hoc fashion based on clinical judgement.

#### Geriatric assessment

Some 95% of MDTs did not have access to prior geriatric assessment, and the method of involvement of a geriatrician was direct referral in 30%. Of these referrals, 55% of MDTs described these as ad hoc (Figure S5a, b and c – available online). The majority of referrals were made postoperatively (Figure S5b – available online).

### Simulated patients

#### Attendance

All MDTs had surgeon and oncologist presence during decision making on the simulated patients and the assessments required to investigate fitness for surgery. CRC specialist nurses (90%), MDT co-ordinator (95%), pathologist (85%) and radiologist (85%) were also in attendance in most MDTs.

#### Assessments for fitness requested

**Figure 4:**
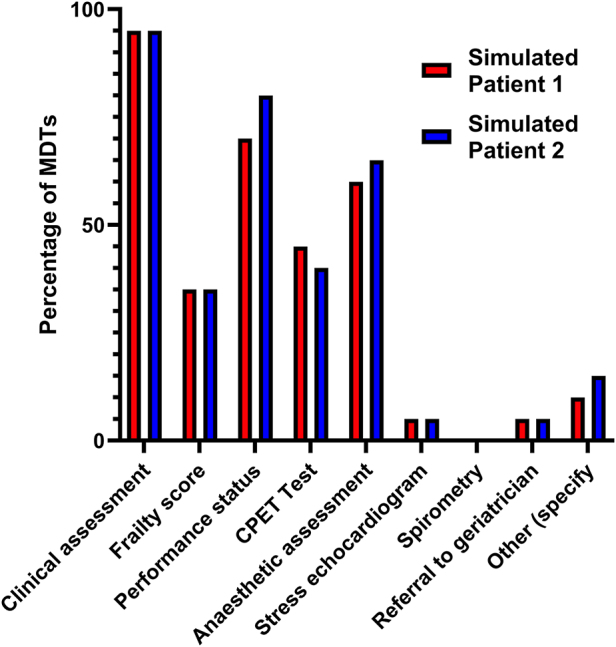
Assessments requested for simulated patients 1 and 2

Figure 4 demonstrates the percentage of different assessments utilised by different MDTs to determine the surgical fitness of the simulated caecal and rectal cancer patients. Clinical assessment was utilised for both patients across all MDTs. WHO performance status was the next most frequently utilised assessment (70% and 80% for caecal and rectal cancer patients, respectively). Frailty scoring was utilised in 35% of MDTs for both patients. There were no distinct differences in utilisation of anaesthetic opinion and CPET between the simulated caecal and rectal cancer patients, respectively: anaesthetic opinion (60% vs 65%) and CPET (45% vs 40%).

#### Treatment decisions

##### Simulated patient 1 (caecal cancer)

The most common recommendation (75%) was primary resection of the tumour. Table 1 outlines the selected treatments and any additional comments.

**Table 1:** Percentage of multidisciplinary team meetings for treatment decision options for simulated patient 1 with caecal cancer

Treatment option	Percentage of MDTs	Percentage of MDTs with additional comments	Additional comments
Resection of primary colon cancer	75	66	60% of MDTs stipulated that surgery required clinical assessment by a consultant surgeon
			50% of MDTs indicated that the decision for surgery would be influenced by both CPET and anaesthetic assessment
Defunctioning stoma	5	0	–
Transfusion	5	5	Requires CPET and anaesthetic assessment for subsequent consideration of surgery
Chemotherapy, radiotherapy, palliative care referral	0	0	–
Best supportive care	5	0	–
Other (customised treatment plans)	15	15	Surgical resection and primary anastomosis after iron infusion, followed by shared decision-making meeting with patient
			Complete colonoscopy for histological diagnosis, followed by clinical and anaesthetic assessments
			Surgery to be considered if anaesthetic opinion deemed patient to be ‘fit’

CPET = cardiopulmonary exercise testing; MDT = multidisciplinary team

##### Simulated patient 2 (rectal cancer)

Decision making showed more variability. Only 55% of MDTs recommended eventual surgery; the rest opted for palliative radiotherapy or other non-surgical approaches. No single treatment option was chosen by more than 20% of MDTs, reflecting a lack of consensus on the best treatment approach (Table 2).

**Table 2:** Frequency of decisions for treatment options for simulated patient 2 with rectal cancer

Treatment option	Percentage of MDTs	Percentage of MDTs with additional comments	Additional comments
Hartmann’s resection	20	15	Begin with an iron infusion Stenting, long- and short-course chemoradiotherapy to be ruled out Surgery to follow pending clinical assessment, if unfit best supportive care was recommended
			Following CPET and anaesthetic assessment a Hartmann’s would be considered
			Not for neoadjuvant treatment
Anterior resection	15	5	Decision between anterior resection or Hartmann’s made in shared decision-making meeting with patient
Palliative radiotherapy	20	10	For radiotherapy if unfit for surgery. If not, decision for surgery in shared decision-making meeting
Short-course radiotherapy and surgery	10	5	Completion colonoscopy prior to radiotherapy. Fitness to be assessed in conjunction with oncologist and MacMillan nursing team
Short-course radiotherapy, delay and surgery	10	0	–
Long-course chemoradiotherapy and surgery	10	0	–
Other	20	20	Unlikely to be a surgical candidate, requires chemoradiotherapy assessment
			Prioritised surgeon’s clinical assessment, anaesthetic assessment and CPET followed by shared decision-making meeting
			If favourable preoperative assessments: treatment will be short-course radiotherapy followed by anterior resection Unfavourable assessments would aim for total neoadjuvant therapy with, potentially a Hartmann’s procedure as an option
			Hartmann’s procedure or anterior resection based on patient fitness. If unfit, options included stent placement, palliative radiotherapy or best supportive care

CPET = cardiopulmonary exercise testing; MDT = multidisciplinary team

### Free-text options

All free text can be viewed in Table S1a and S1b (available online). Some free-text phrases illustrating difficulty in decision making were: “If fit, then Hartmann’s or anterior resection … . If not fit, stent/palliative radiotherapy/best supportive care”. Another example was, “This is tricky …  If fit enough we might go for a Hartmann’s, if not, palliative radiotherapy or best supportive care might be best option”.

Throughout, the free text provided a continual theme of understanding patient choice given the risk of scenario was consistently emphasised. Such phrases included, “Patients wishes and their willingness to accept risk of surgery” and “ … discussion with patient about treatment options and their feelings on proceeding v no treatment” as well as “Discussion with patient–patient choice”.

## Discussion

This study revealed significant variation in both MDT resources and decision making for simulated CRC cases (rectal vs caecal cancer resection). These findings collectively illustrate the complex landscape in which MDTs operate, leading to differences in patient outcomes.

A key finding was the dichotomy in MDT approaches. Approximately half of the MDTs made collective decisions, whereas the other half deferred primarily to the named clinician, who was supported by the MDT. This divergence suggests a broader question regarding the MDT’s role in decision making. Ideally, a consensus MDT plan should guide the surgeon; but in practice, the named clinician often makes the final decision, sometimes encountering the patient for the first time post-MDT without prior knowledge of the patient’s frailty. This leads to frequent changes in treatment plans, affecting up to 52% of cases post-MDT, as noted in previous reports.^11–15^ Because frailty screening is embedded across National Health Service (NHS) services, such as emergency department visits for adults aged 65 years and older, this could be extended to the colorectal MDT.^16^ A potential solution could be standardised questionnaires administered by junior team members or allied health professionals to gather critical information on frailty, as previously piloted in a UK study.^17^

CPET use varied substantially between centres. Although associated with favourable outcomes in some studies, CPET primarily assesses cardiorespiratory fitness rather than overall frailty, making it more appropriate for younger patients with comorbidities.^18–20^ Other assessments like frailty scoring or sarcopenia measurement may be more suitable.^21^ Inconsistent CPET use may reflect a limited understanding of its role in this demographic or a lack of anaesthetic input in MDTs.

Geriatrician involvement in MDTs was minimal, usually occurring postoperatively through ad hoc referrals, aligning with existing literature, despite strong evidence of the benefit of preoperative assessment in other surgical fields like orthopaedic and vascular surgery.^22–25^ In addition, routine, rather than ad hoc geriatrician involvement is recommended both nationally (BGS) and internationally (ASCRS).^9,26^ Given the increased complexity in decision making with frailty, comorbidities and cognitive impairment, routine preoperative geriatrician input and attendance could enhance the MDT as recommended by the National Emergency Laparotomy Audit and the American College of Surgeons Geriatric Surgery Verification Program.^27–31^

Although incorporating routine preoperative geriatric services and, incorporating Comprehensive Geriatric Assessment (CGA) would ideally be implemented in the future of major elective abdominal surgery, its implementation is problematic owing to disparities in the availability of geriatricians across different regions.^32,33^ As a viable alternative, triaging patients using the G8 screening tool acknowledged as an optimal tool to identify patients for oncogeriatric evaluation, could benefit from CGA. This could screen patients prior to MDT discussions, as it has been shown to improve outcomes in frail patients undergoing adjuvant chemotherapy, highlighting the need for targeted geriatric involvement.^34,35^

Triaging patients using the G8 tool is a potential strategy; however, using clinical frailty scoring (CFS), which is established to affect shared decision making, can predict and improve outcomes for elective colorectal cancer resections in an octogenarian population.^24^ Despite this, our results demonstrate its underutilisation, owing to either limited awareness or limited capacity in the NHS to identify and manage frailty.^36,37^ Therefore, the implementation and integration of such changes to the MDT pathway are not straightforward owing to the previously mentioned infrastructure and disparities in geriatrician staffing.

Our study found that half of the MDTs recommended surgical treatment for the rectal cancer patient, with fewer planning an anastomosis, whereas 75% opted for a caecal resection with primary anastomosis. This likely reflects the higher complication risk with anterior resection, where morbidity from pelvic dissection is higher and anastomotic leaks are more frequent, especially with lower anastomoses, despite its superior overall survival.^38–45^ Given these risks and the patient’s frailty, there may be less appetite for rectal resections with primary anastomosis. Interestingly, a similar number chose Hartmann’s procedure, possibly because of comparable operating times and the inherent risks of pelvic surgery. These findings highlight the considerations in surgical assessment and the careful balancing of benefits and complications during treatment planning.

The differing management approaches for rectal and caecal cancer patients could be attributed to perceived surgical risks, available treatment options and patient frailty status. Whereas caecal tumour management typically involves a binary choice between surgery and no treatment, rectal cancer offers multiple, albeit non-gold-standard, options. This flexibility might lead MDTs to favour less-risky, non-curative approaches in frail patients. In addition, the availability of neoadjuvant and palliative regimes for rectal cancer contrasts with the limited options for caecal cancer, influencing MDT decisions.^46^ This variety in treatment possibilities underscores the importance of personalised care plans tailored to patient-specific factors.

Surgeon personality could explain the differences in risk appetite for management of the rectal cancer patient. The PLATO project recently identified that different surgeon personality profiles are an independent factor in deciding whether to form an anastomosis or exteriorise the bowel as a stoma.^47^ Traits such as agreeableness, extraversion and openness were quantified. Surgeons with higher levels of openness when providing a second opinion were associated with a higher rate of stoma formation. Prior clinical experience throughout surgical training and independent practice can shape a surgeon’s acceptability of risk because of responses to successful and unsuccessful outcomes. This may influence how surgeons make decisions concerning higher-risk rectal cancer patients. In addition, many colorectal surgeons will not have formally trained in frailty assessment; thus, it is difficult to make these decisions when left to the individual surgeon. Therefore, variation in practice can be partly attributed to this lack of training.

The free-text responses highlighted the importance of shared decision making. Shared decision making within MDTs for CRC management should be a collaborative process that engages all team members, rather than placing the burden solely on the treating surgeon or the patient. To achieve this, routine or selective involvement of geriatricians in the MDT (as previously mentioned), using CGA, could distribute decision-making responsibilities more evenly and provide a holistic evaluation of patient needs. In addition, incorporating preoperative anaesthetic clinics would further support an MDT-driven shared decision-making approach by preparing patients comprehensively before surgery. To ensure active patient involvement, simplified yet comprehensive case summaries could be provided, facilitating understanding of the input of MDT members. MDT-developed patient decision aids would facilitate clear communication and support informed shared decision making, and have already been successfully implemented by NICE.^48,49^

### Study limitations

There are limitations in this study; first, the limited sample size of 20 UK MDTs, which may not be representative of worldwide colorectal practice. Second, there is regional bias, as shown in Figure 1: the majority of MDTs responding were from northwest England. Third, although the survey and hypothetical scenarios were designed to capture a range of responses, the free-text options demonstrate the extent of decision-making variability. Therefore, qualitative interviews with MDT members as well as live assessment of the MDT discussion may provide deeper insights into the decision-making process.

## Conclusion

In conclusion, this study highlights the complex decision-making processes within MDTs for frail patients undergoing CRC treatment, revealing significant variations in resource availability and approaches. The differences in decision making between rectal and caecal cancer patients illustrate the challenges of surgical risk assessment, especially in the context of frailty. The underutilisation of assessment tools indicates a gap between evidence-based practices and their clinical application to implement such tools. Solutions include implementation of CFS at the time diagnosis for early identification of frail patients. This could be supported by the development of a frailty assessment pathway incorporating a standardised set of resources (geriatrician input, CPET), available to the individual centre, prior to the MDT or decision-making meeting to ensure consistent assessments.

Further research is required to enhance decision making and CRC outcomes in frail patients. A potential approach could involve piloting the routine inclusion of geriatricians in MDT meetings, supported by targeted CGA as well as anaesthetists or the aforementioned frailty assessment pathway. This pilot would assess treatment outcomes such as decisional regret to improve standardisation and the quality of MDT pathways as well as the quality of clinical decision making following the integration of geriatric expertise into the care pathway. This approach could be evaluated in terms of both clinical outcomes and resource implications, including its impact on length of hospital stay, treatment tolerance and overall patient recovery, to determine the potential benefits of integrating geriatric and anaesthetic expertise into standard care pathways.
